# Supplementary material for: Trehalose and tardigrade CAHS proteins work synergistically to promote desiccation tolerance
Source: Commun Biol. 2022 Oct 1;5:1046. doi: 10.1038/s42003-022-04015-2 (PMC9526748; doi:10.1038/s42003-022-04015-2)
Supplement: Supplementary file 3 — Description of Additional Supplementary Files [file 42003_2022_4015_MOESM3_ESM.pdf]

Description of Additional Supplementary Files

File name: Data S1.zip

Description: File containing all source data for graphs and other analysis.

File name: Data S2.zip

Description: File containing all code used in this project.
