# Supplementary figures and images for: Trehalose and tardigrade CAHS proteins work synergistically to promote desiccation tolerance
Source: Commun Biol. 2022 Oct 1;5:1046. doi: 10.1038/s42003-022-04015-2 (PMC9526748; doi:10.1038/s42003-022-04015-2)

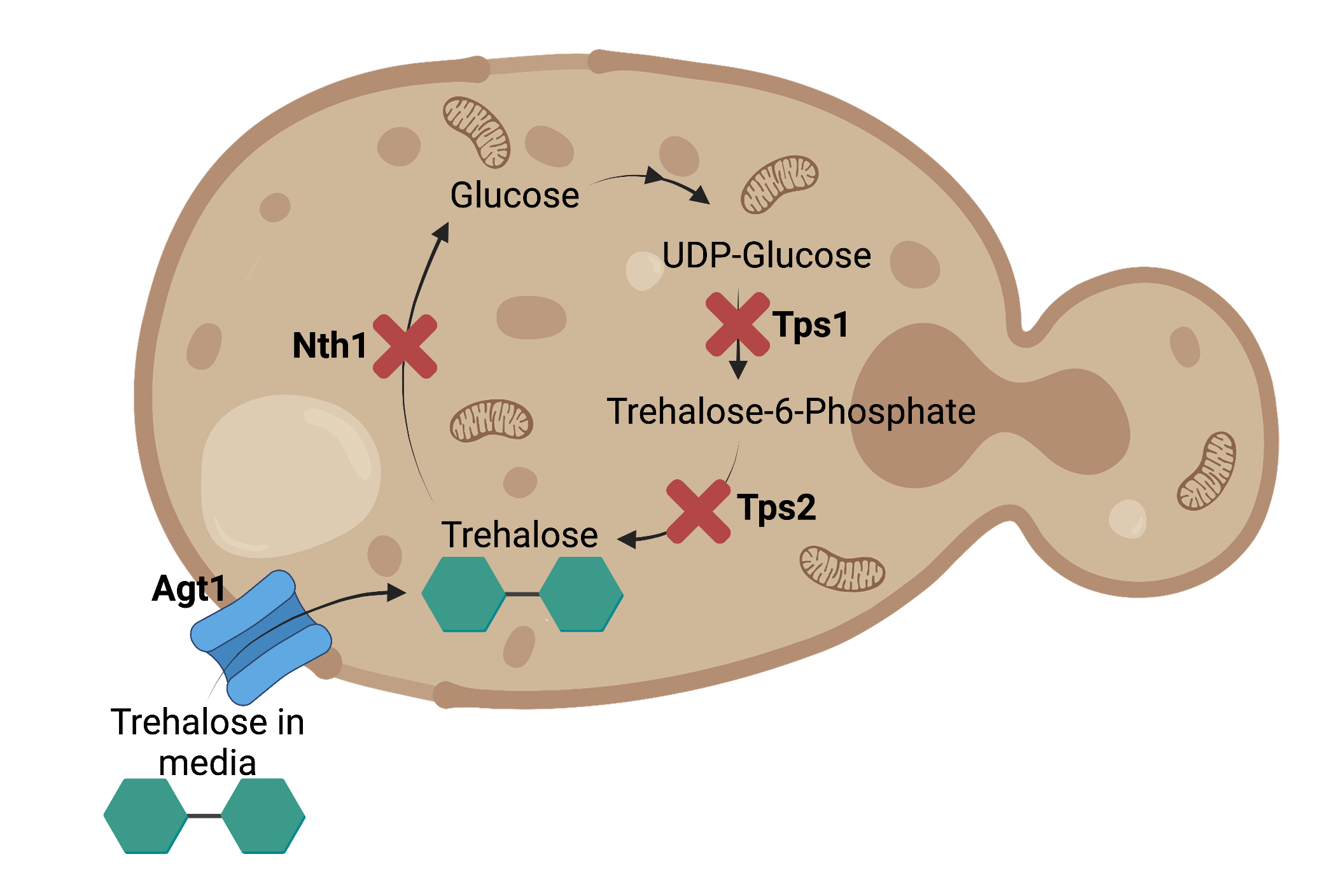

Supplement: Supplementary file 5 — Data S1 [file 42003_2022_4015_MOESM5_ESM.zip › File S1 - Data/Fig. 5 Data/Yeast trehalose_new.tif]

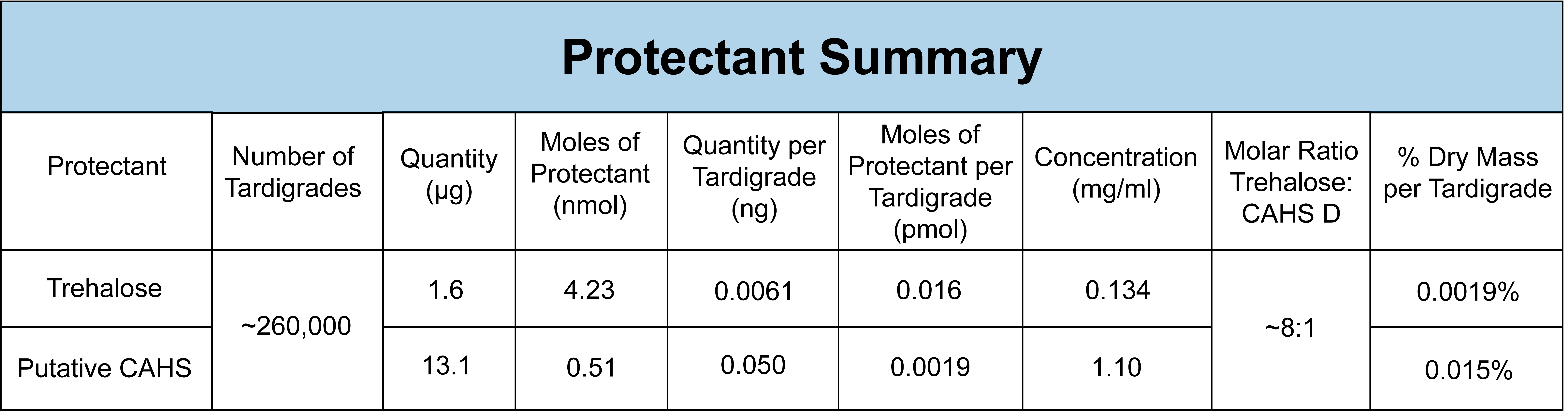

Supplement: Supplementary file 5 — Data S1 [file 42003_2022_4015_MOESM5_ESM.zip › File S1 - Data/Table 1 Data/Table 1.png]
